# Adsorption of Rose Bengal dye from waste water onto modified biomass

**DOI:** 10.1038/s41598-023-41747-5

**Published:** 2023-09-07

**Authors:** Mohammed G. Hassan, Magdy A. Wassel, Hosni A. Gomaa, Ahmed S. Elfeky

**Affiliations:** Chemistry Department, Faculty of Science, Al-Azhar University, Nasr City, Cairo, 11884 Egypt

**Keywords:** Chemistry, Analytical chemistry

## Abstract

Herein, adsorption of Rose Bengal dye (RB) from aqueous solution was investigated. Nano raw orange peel (OP) activated carbon (AC) coated with nano chitosan (Cs) to obtain nano chitosan/activated carbon (AC/Cs) composite which cross-linked with functionalized multi-walled carbon nanotubes (MWCNTs-COOH) to create a novel composite (AC/Cs/MWCNTs) with high surface area (1923 m^2^/g). The examined parameters such as concentration (1–7 ppm), pH (6.5–9.5) and temperature (295–323 K) were traversed. The maximum removal efficiency was at pH 6.5, increased from 70.4% for nano OP to 94.7% for AC/Cs/MWCNTs nano composite. Langmuir isotherm model was the best fitting to acquired data (R^2^ ≥ 0.99). Also, the adsorption of RB matched with pseudo-second order model, t^0.5^ results for pseudo-second order was 4.4672 for nano OP and 1.2813 for AC/Cs/MWCNTs at 303 K. Thermodynamic studies showed that the adsorption of RB dye is exothermic and spontaneous due to the negative value of ΔG and ΔH.

## Introduction

Nevertheless, urban and population development were and remain among the most important causes of the global water system disruption. This distribution has made people eager to find viable solutions to restrict the clear decline in water supply. Therefore, strenuous efforts have been made to treat the industrial wastewater which releases different types of pollutants such as phosphor, fertilizers, pesticides, heavy metal and dyes^1–4^.

Dyes are one of the most important categories of pollutants which defined as aromatic, colored, and ionizing substances. Coal tar-based hydrocarbons are the origin of dyes, such as benzene and xanthene which used to color different substrates, such as drugs and textile which utilize about 7 × 105 tons of dyes annually. Removal of dyes from wastewater receive widespread attention owing to their high toxicity, low biodegradability and innumerable problems the aquatic system and also the human health^5^. Among these dyes Rose Bengal (RB), it follows xanthene dyes species, known as C.I. 45440 and C.I. Acid Red 940, is chemically referred as disodium-4,5,6,7-tetrachloro-30,60-dihydroxy-20,40,50,70-tetraiodo-3H spiro [isobenzofuran-1,90-xanthen]-3-one (molar mass: 1017.64 g/mol). RB dye sodium salt is frequently used to diagnose eye injury by staining corneal and conjunctival cells, as well as to treat some malignancies. Even though, these advantages, it has a close connection to various eye and skin conditions^6,7^.

Wastewater treatment techniques varied between different methods including adsorption, coagulation, and photo-catalytic degradation. Of these, the preference was for adsorption, due to its high efficiency, cheap cost, feasibility, design simplicity and non-generation of toxic materials^8–12^. Various adsorbents such as raw agricultural wastes such as raw orange peel^13^, activated carbon^14^, polymers such as chitosan (Cs)^15^, carbon nanotubes^16^ and cross-linked composites^17^ have been broadly used to treat dye-contaminated wastewater.

Over the last few years, agricultural waste received unprecedented attention in wastewater treatment for its great effectiveness in the adsorption process^18^. Based on FAO reports, the generation of orange peel wastes is nearly in the range of 15–25 million tons. Dumping of these wastes is a very difficult process. Generally, Orange peel contains several unique components such as cellulose, hemicelluloses, pectin (galacturonic acid), lignin and chlorophyll pigments. These different components can be effectively contributed to treatment of dye-contaminated water since they have a variety of functional groups, including amide, carboxyl, and hydroxyl^19^. Activated carbon has several exceptional features that have made him find a prestigious place among its peers and made it the most common adsorbent for treatment of dye contaminated water. These features revolve around its adsorption great capacity, large surface area, mighty degree of surface activity, and its microporous structure^20^. Much effort is currently being paid to the production of ACs from agricultural residues due to the rising need for inexpensive ACs, especially those derived from renewable sources^21,22^. Chitosan (Cs) biopolymer is a multifunctional cationic biodegradable polysaccharide produced by deacetylation of chitin. It is one of the most-used biological molecule in the adsorption of various classes of water pollutants due to its non-toxicity, biodegradability, biocompatibility and its richness in reactive functional groups such as OH and NH_2_ groups^23^. The dye adsorption mechanism on Cs-based biomaterials is primarily controlled by n–π interactions, electrostatic interaction, and hydrogen bonding. As a result, by adding functional groups like aromatic rings on the skeleton of the Cs, an aromatic system of the fabricated material could interact with the aromatic system of dyes π–π stacking, boosting the removal effectiveness of organic dyes^24^. The great efficiency of multi-walled carbon nanotubes (MWCNTs) in dyes removal is imputable to electrostatic interaction, H-bonding, hydro-phobic effect, and π–π stacking^25,26^. MWCNTs functionalized (H_2_SO_4_/HNO_3_) introduce oxygen-containing groups on MWCNTs surface, which definitely enhance their hydrophilicity, enhance its chemical activity, exfoliate the CNTs bundles, and enable their dispersion in polar media^27–29^.

AC plagued by some precarious deficiencies that mitigate its application: its powdery form, particle size smallness, complexity of regeneration likewise, the difficulty of its isolation from aqueous solutions were the most prominent factors that limit AC usage on its original form. On the other hand, the powder form of raw Cs causes a great difficulty in its separation from aqueous solutions after completion of the adsorption process. Cs crystal form and its hydrophobic features Its crystallinity and hydrophobicity reluctant the rate of liquid–solid mass transfer and causing column obstruction and high pressure drops, which in turn leads to high operation costs^30^. The small particle size of CNTs caused high difficulty to separate it from the aqueous media after the adsorption operation completed. It has been found that the new trend in of adsorption efficiency improving is the chemical modification^31,32^. Combination of Cs and AC can improve their thermochemical and mechanical properties and overcome their shortcomings which thus, increase their precious for practical applications^33–35^. Also, Cs can be used to modify the surface of carbon nanotubes CNTs, this in turn, has a lot of features such as dispensability enhancement, substrate binding ability, shortened adsorption time and increase adsorption capacity, improvement the surface morphology and the chemical composition becomes well than before^36–38^.

## Materials and methods

### Materials

All chemicals/reagents used in this study were analytical grade. (O Phosphoric Acid, 85% H_3_PO_4_) purchased from Fisher scientific, Cs (85% analytical grade from shrimp shells), Dodecyl sodium sulfate (SDS) CH_3_(CH_2_)_11_OSO_3_Na, gluterdehyde and MWCNTs-COOH were supplied by Sigma-Aldrich. RB dye, glacial acetic acid obtained from Merck.

### Instruments

The portable FT-IR provided by Bruker Optik GmbH was used to detect the functional groups present in the prepared adsorbents in the spectral domain ranging 400–4000 cm^−1^ at room temperature. XRD-6100 X-ray diffractometer was used to perform the X-ray diffraction patterns for the adsorbents with CuKα (λ = 1.5406 Å) radiation 2θ range of 10°–90°. The continuous scanning mode was in the scan speed2deg/min, the sampling pitch was 0.02° also, the preset time was 0.6 s. The morphology of the prepared adsorbents was analyzed using a scanning electron microscope (SEM, Jeol JSM-6510 LV) with Energy Dispersive X-ray Spectrometer (EDX). Transmission electron microscope (TEM) patterns were obtained by JEM 2100, JEOL. Shimadzu spectrophotometer (UV-1601 PC) with the matched cells 10 mm was used to measure the absorbance values. NOVA e-Series analyzer was applied for measuring the BET surface area of samples.

### Preparation of RB stock solutions

Various stock solutions of analytical reagent grade RB dye (1 ppm, 3 ppm, 5 ppm and 7 ppm) were prepared with dissolving the desired amounts in deionized water. Batch experiments were proceeded using the diluted dye solutions. The pH values of the prepared solutions were adjusted using 0.1 mol L^−1^ CH_3_COOH or NaOH solutions.

### Preparation of nano OP

Orange Peels (OP) were purchased from a local fruit market, washed with tap water then hot deionized water for several times to remove dust and other contaminants. Clean peels dried at sunlight for 48 h. The dried orange peel cut into tiny pieces and dried in vacuum oven at 70 °C during 4 h until completely drying. The dried samples were milled and screen-sieved using 0.355 mm mesh. Finally fine particles milled in a homogenizer ball mill for 1 h until size (50–100 nm).

### Preparation of nano AC

OP sample was carbonized up to 420 °C at a heat rating 7 °C/min for 1 h. 1 g of carbon mixed with 85% wt H_3_PO_4_ solution was and placed in a muffle at 600 °C during 1 min per gram of sample. After the sample cooled to room temperature, deionized water was used to attained neutral pH. The sample dried in a vacuum oven at 80 °C during 6 h therefore, nano orange peel activated carbon (AC) was obtained.

### Processing of nano Cs

Nano Cs washed by doubly deionized water until pH 7 then dried in vacuum oven at 50° for 4 h. 1 gm of nano Cs dispersed into 50 mL of 0.5% vol. acetic acid. The colloidal stirred for 24 h until homogeneous gel formed.

### Preparation of nano AC/Cs

4 g of nano AC Added to 1 gm of nano Cs gel and stirred for 12 h at 150 rpm. The mixture dried at 60 °C for 3 h to obtain Cs thick layer on AC. Activation and neutralization carried out by soaking of the mixture in 75 mL (0.5% wt./v) of NaOH during 3 h, rinsed with deionized water until neutralization and dried at 80 °C for 12 h under vacuum.

### Preparation of AC/Cs/MWCNTs nano composite

4 g of nano AC/Cs composite added to 100 mL of (1%) acetic acid then stirred for 1 h. This mixture put in a dry clean bottle named bottle 1. With the assistance of SDS as a dispersant, 5 ml of SDS put in 10 mL of deionized water, pH of the solution neutralized at pH 7 using NaOH (0.1 M), 3 mL of this solution put in a dry clean bottle and 0.75 gm of MWCNTs-COOH added to this bottle then stirred for 1 h, this bottle named bottle 2. Bottle 1 added to bottle 2 and put under ultrasonic for 1 h. 50 mL of (4%) NH_4_OH dropwise added into the solution. The suspension heated at 60 °C under stirring then 0.2 g of gluterdehyde added into the system. The suspension stirred without heating for 12 h then rinsed with doubly deionized water and dried under vacuum at 50 °C for 80 h to obtain the final AC/Cs/MWCNTs nano composite.

### Adsorption experiments

It is believed that the equilibrium isotherm is considered a critical point in diagnosing the adsorptive characteristics for the adsorbent under consideration, this in turn contributes to knowing the right direction of the adsorption process design. Batch experiments were carried out via adding steady amounts (0.05 g) of nano OP or AC/Cs/MWCNTs nano composite powders to a series of 50 mL beakers, containing RB dye solutions with a desired concentration in ppm. The prepared mixtures stirred using a magnetic stirrer at 150 rpm for 120 min at room temperature (295 K) for equilibrium state achievement. Then, an amount of each sample withdrawn from the stirred solution at different timings, filtrated well from any adsorbent residues and put into tightly closed dry clean bottles marked with the time at which the sample was withdrawn, spectrophotometer instrument used to measure the absorbance values at 540 nm. The mass balance equation present in (Eq. 1) is also used to calculate the adsorption capacity (q_e_) of RB dye that adsorbed on the mass unit of nano OP or AC/Cs/MWCNTs nano composite in ppm: Where V is the volume of RB dye solution in liter, C_0_ refers to the initial concentration and C_e_ are and equilibrium concentrations of RB dye in ppm per litter and m is the amount of nano OP or AC/Cs/MWCNTs nano composite used in grams. Freundlich and Langmuir were the two models used to investigate the equilibrium adsorption of RB dye from aqueous medium Additionally, pseudo first order, pseudo second order and intraparticle diffusion were the models used for calculation of kinetic parameters of RB dye adsorption.

1 $${q}_{e =\frac{\left( {C}_{0}-{C}_{e}\right)V}{m}}$$

## Results and discussions

### Characterization of adsorbents

#### FT-IR spectra

**Figure 1:**
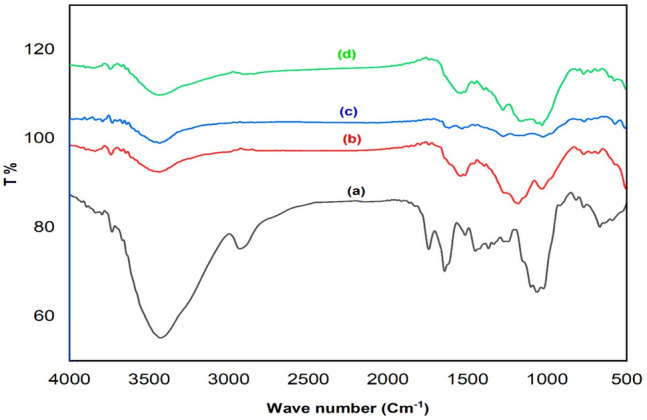
FTIR for (**a**) nano OP, (**b**) nano AC, (**c**) nano AC/Cs and (**d**) AC/Cs/MWCNTs nano composite.

Figure 1 showed the FTIR results for nano OP, nano AC, nano AC/Cs and AC/Cs/MWCNTs nano composite. In nano OP FTIR (Fig. 1a), a large peak appears at the wavelength 3430 cm^−1^ related to the stretching vibration of O–H group that owing to the inter and intra molecular hydrogen bonding of alcohols, carboxylic acids and phenols in orange peel components such as cellulose, pectin and lignin. After the carbonization the absorption peak shifted to larger wavelengths by nearly 8 cm^−1^ to assigned the existence of O–H groups on its free form after carbonization as shown in (Fig. 1b). After Cs grafted AC, this peak is shifted to higher frequency to become 3445 cm^−1^ which may assigned to that both amine (NH) and hydroxyl (OH) groups of Cs is assigned to overlapping of the stretching vibrations. The same peak appears in the AC/Cs/MWCNTs nano composite FTIR to give clear evidence the successful of all modification processes as revealed in (Fig. 1c). The peak at 2931 cm^−1^ which appears at nano OP FTIR (Fig. 1a) belongs to C–H stretching vibration of methyle, methylene and methoxy groups in nano OP components. This sharp peak shifted slightly to 2908 cm^−1^ in AC/Cs/MWCNTs nano composite FTIR (Fig. 1d) due to of C–H stretching related to aldehydes, this evidencing the successful modification of MWCNTs-COOH with nano AC/Cs composite, the same stretching vibration of these groups in Cs. A broad peak appears at 1276 cm^−1^ in FTIR of AC/Cs/MWCNTs nano composite (Fig. 1d) citing the C–O–C stretching which denote the crosslinking reaction of glutardehyde which enhance the interaction between the three materials through –OH crosslinking of these materials.

#### BET

**Figure 2:**
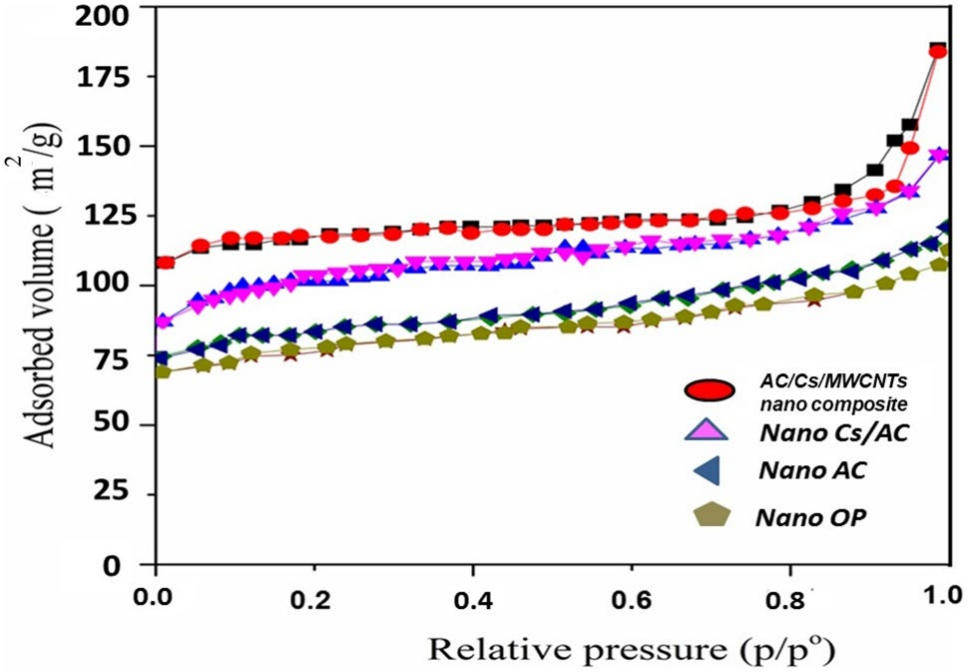
BET for (**a**) nano OP, (**b**) nano AC, (**c**) nano AC/Cs and (**d**) AC/Cs/MWCNTs nano composite.

BET results are shown in Table 1 and Fig. 2. Our results revealed that the BET surface area of nano OP is 289.720 m^2^/g. The evaluated average pore size confirms that the material is mesoporous. Therefore, the mesoporous material has a better advantage over the raw nano OP. The specific surface area of nano AC is 1398 m^2^/g. The pore structure of nano AC is mesoporous. Mesoporosity reached 68.9% all of this pointing to a pronounced pore development during the treatment. As reported for the activation process of other lignocellulosic precursors, the H_3_PO_4_ acid reacting with the internal cellulose structure induces a depolymerization leading to an enhancement of the pore volume and, thus, a global volume expansion. The BET surface area of AC/Cs/MWCNTs nano composite is 1923 m^2^/g. The larger surface area of the AC/Cs/MWCNTs nano composite sorbent provided more adsorption sites that increased adsorption capacity and improved extraction efficiency after modification processes. The micropore volume fraction is higher than 85%, confirming the majority of micropores.

**Table 1:** BET analysis for RB dye on nano OP and AC/Cs/MWCNTs nano composite.

Textural properties	Nano OP	Nano AC	AC/Cs/MWCNTs composite
S_BET_ (m^2^/g)	289.720	1398	1923
V_t_ (cm^3^/g)	0.1821	2.152	0.181
V_μ_ (%)	2.321	18.7	9.2
W (nm)	5.732	4.543	0.875

#### X-ray diffraction

**Figure 3:**
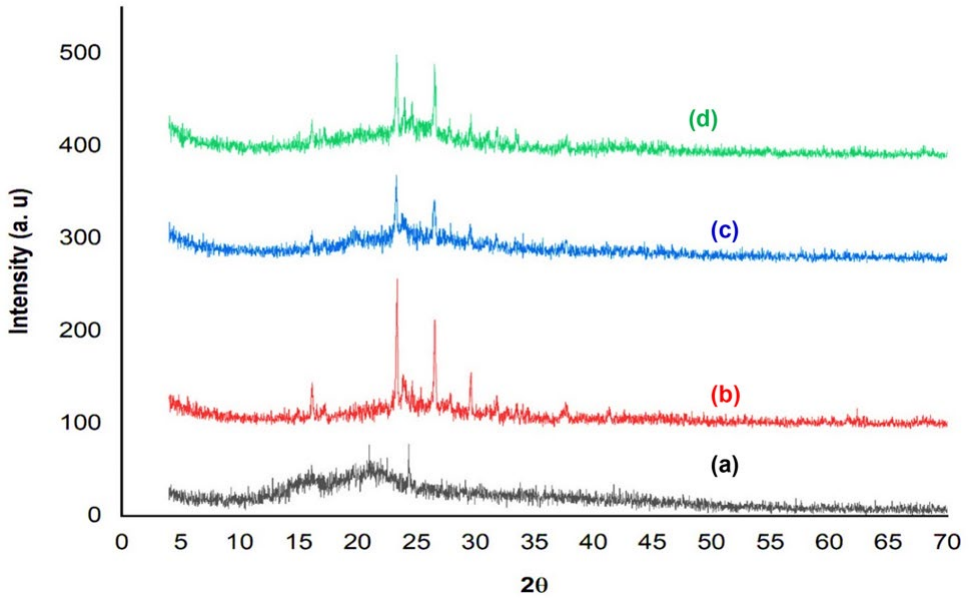
XRD for (**a**) nano OP, (**b**) nano AC, (**c**) nano AC/Cs and (**d**) AC/Cs/MWCNTs nano composite.

Figure 3 represents XRD patterns for nano OP, nano AC, nano AC/Cs and AC/Cs/MWCNTs nano composite. The XRD spectrum of nano OP (Fig. 3a) showed that there are no any well-defined bands in any area and therefore, no detection of any distinct mineral phase. Subsequently, the nano OP has an amorphous structure entirely, as ever for the organic materials. The XRD diagrams of nano AC (Fig. 3b) reveals the broad main peak assigned to the existence of crystalline highly structured of carbon of rhombohedral. It is noteworthy that the crystalline structure enhances the adsorption efficiency of dyes on the prepared nano AC. Broadness and intensity of peaks are a clear demonstration that the existence of carbon structure regarding the range of degrees, referring to the carbon crystalline form present in nano AC. After AC/Cs decoration then loading with MWCNTs-COOH, it was noticed that the intensity of peaks of nano AC were reduced in intensity as shown in (Fig. 3c,d). It is recalled that this amorphous phase is the most optimal orientation for adsorption because of the active sites are more affordable for adsorbate.

#### SEM/EDS analysis

**Figure 4:**
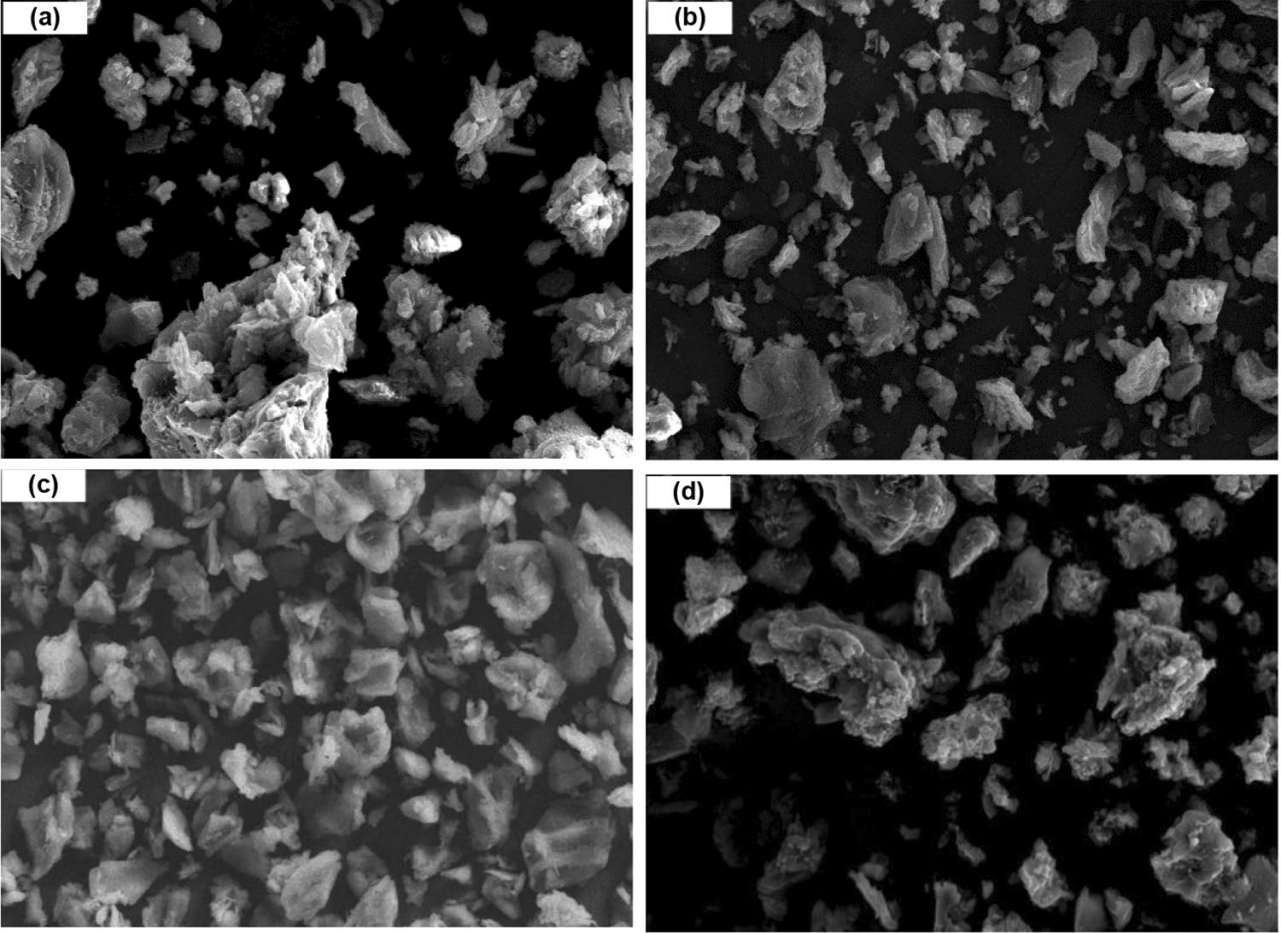
SEM images for (**a**) nano OP, (**b**) nano AC, (**c**) nano AC/Cs and (**d**) AC/Cs/MWCNTs nano composite.

The morphology of the nano OP, nano AC, nano AC/Cs and AC/Cs/MWCNTs nano composite samples are shown in SEM images Fig. 4. Nano OP SEM (Fig. 4a) shows flattened walls and large irregular intercellular spaces with different sizes and shapes which due to the presence of different components that consist the peel such as cellulose, hemicellulose, pectin and lignin. Nano AC SEM (Fig. 4b) shows conducts of different shapes and sizes with interconnected micro pores channels which due to the rearrangement of the structure of nano OP during the activation process. Evident cavities were observed on the surface of nano AC/Cs SEM (Fig. 4c) which confirm the successful of Ac grafting on Cs surface. AC/Cs/MWCNTs nano composite SEM (Fig. 4d) shows that the surface become neat and smooth. A little agglomeration appears due to the nano Cs polymer viscidity. The distribution of MWCNTs-COOH decorated by nano Cs and nano Ac is more symmetrical which expressed that the crosslinking process.

EDS analysis before adsorption for nano OP, nano AC and AC/Cs/MWCNTs nano composite and after adsorption for nano OP and AC/Cs/MWCNTs nano composite has been represented in the and Table 2. The percentage weight of chemical compositions available on the surface of nano reveals the existence of proteins and polysaccrides inside the biomass cell cover. Nano AC EDS shows that the nano AC is constituted of several elements, such as: magnesium, phosphorus, surfer, chlorine, potassium, calcium, and iron. Most of these elements are a characteristic of the orange peel composition. The presence of oxygen is an indication that the nano AC had a lot of oxygenated groups on its surface. The results of the EDS analysis of AC/Cs/MWCNTs nano composite shows that it is consist of mainly carbon (C) and oxygen (O). The increase of carbon content in the AC/Cs/MWCNTs nano composite was consistent with the added amount of CNT. Nano OP and AC/Cs/MWCNTs nano composite after the adsorption of BR dye contain greater C and O content due to the structure of the dye, which is rich in both elements. Also, the two adsorbents have accumulated other trace elements present in the aqueous dye solution.

**Table 2:** EDS analysis for RB dye on nano OP and AC/Cs/MWCNTs nano composite.

	Wt. %	C	O	N	K	Ca	Mg	Na	AL	Si	P	S	Fe	Cl
Before adsorption	Nano OP	34	45.62	–	12.52	0.35	3.80	1.33	1.31	1.07	–	–	–	–
	Nano AC	63	18.77	–	6.4	8	0.4	–	–	–	1.4	0.52	0.31	0.2
	Composite	78.71	11.26	10.03	–	–	–	–	–	–	–	–	–	–
After adsorption	Nano OP	44.22	54.13					0.86				0.04		0.75
	Composite	83.37	12.75	2.41				0.36				0.13		0.98

#### TEM analysis

**Figure 5:**
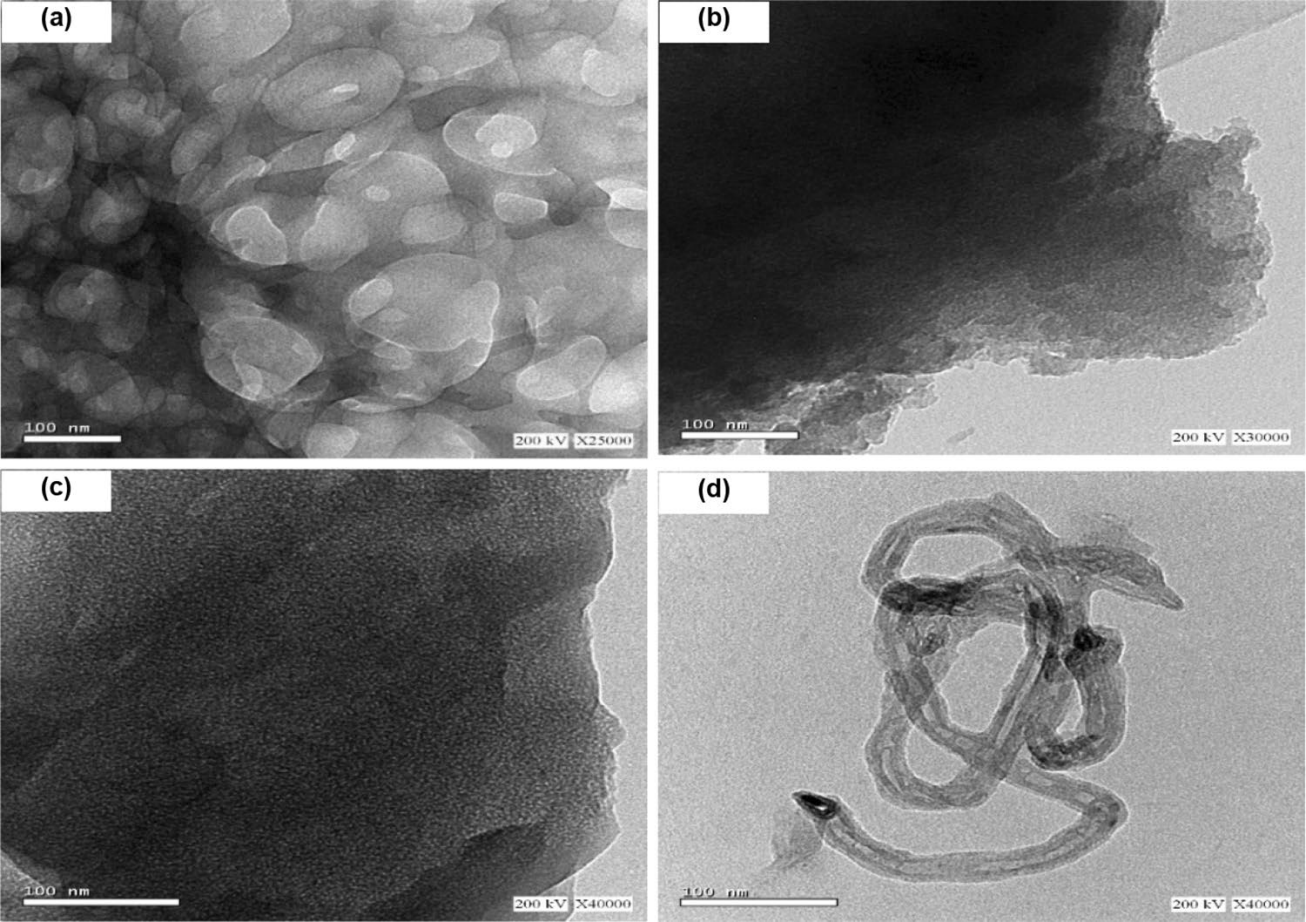
TEM analysis for (**a**) nano OP, (**b**) nano AC, (**c**) nano AC/Cs and (**d**) AC/Cs/MWCNTs nano composite.

The microstructure of the nano OP, nano AC, nano AC/Cs and AC/Cs/MWCNTs nano composite are shown in Fig. 5. The TEM images clearly indicate that the nano OP (Fig. 5a) has achieved significant improvement in surface characteristics as most of the particles ranging from 50 to 100 nm in size. After activation (Fig. 5b) we can notice that the nano AC has rich mesoporous and the surface became not blocky which in role enhance the adsorption process. After nano Cs and nano AC decoration (Fig. 5c) we can notice that the surface become more porous and we can notice the well dispersion of nano AC on Cs surface with more contrast shape. MWCNTs-COOH have a strong tendency to form bundles due to the viscous Cs on the surface. The TEM image for AC/Cs/MWCNTs nano composite (Fig. 5d) exhibits a diameter in the range of 50–100 nm with the increase of the wall thickness confirming the decoration of chitosan on carbon nanotubes.

### Batch adsorption experiments for RB dye removal

#### Effect of RB dye concentration on adsorption process

**Figure 6:**
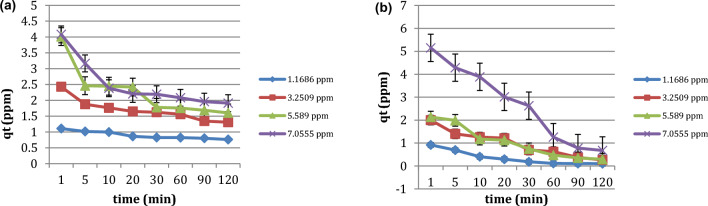
Effect of initial concentration of RB dye on adsorption capacity of (**a**) nano OP and (**b**) AC/Cs/MWCNTs nano composite.

Figure 6 shows the removal efficiency of RB dye by nano OP and AC/Cs/MWCNTs nano composite at various concentrations ranges from1 to 7 ppm. Batch experiments were proceeded by adding a 0.05 g each of two materials individually to 50 mL of various concentration of RB solution (1–7 ppm). The experiments were operated at surrounding temperature of 295 K for 120 min for investigating the effect of variance initial RB concentration on the adsorption effectiveness. In case of nano OP, as the RB equilibrium concentration increases (from 1 to 7 ppm), as the adsorption capacity increased. This removal percentage for the nano OP was 34.7, 59.7, 71.3, and 72.8%, for concentrations 1 ppm, 3 ppm, 5 ppm and 7 ppm, respectively. This in role means presence more of vacant binding sites on the surface of nano OP material to adsorb more of RB dye. On the other hand, in case of AC/Cs/MWCNTs nano composite, the situation is slightly different, as the removal percentage of RB dye on the surface of AC/Cs/MWCNTs nano composite was 91.4, 92, 94.7 and 90.3% for concentrations 1 ppm, 3 ppm, 5 ppm and 7 ppm respectively. We can see obviously that the removal percent of RB dye increases with of the concentration from 1 to 5 ppm. An opposite trend appears when the concentration increases to 7 ppm, the removal percentage decreases to (90.3%). This suggest that the AC/Cs/MWCNTs nano composite can remove about 94% at the concentrations below 7 ppm. In fact, raising the initial RB dye concentration causes an increase in adsorption capacity, which creates a driving force between the adsorption medium and the adsorbent to get past the resistance of mass transfer. As the initial ion concentration increases, the adsorbent's ability to remove ions increases. The ions interact with the binding sites when the concentration of ions is low in the solution, which causes the removal percentage to increase. However, due to saturation of the binding sites at greater concentrations, additional ions may stay in the solution medium without being adsorbed^39^.

The maximum adsorption efficiency of other adsorbent for RB dye is shown in Table 3. As it is seen in Table 3, the maximum adsorption efficiency of AC/Cs/MWCNTs nano composite is higher than several other adsorbents. The comparison of adsorption efficiencies indicates that AC/Cs/MWCNTs nano composite exhibited a reasonable efficiency for RB dye adsorption from aqueous solutions.

**Table 3:** Adsorption efficiency of different adsorbent for RB dye adsorption.

Adsorbent	Adsorption efficiency %	References
AC/Cs/MWCNTs nano composite	94.7	Current work
Chitosan/Zirconium oxide nanocomposite	78	^40^
Chitosan blended polyacrylonitrile	83	^41^
Green and sustainable Chitosan/CMC/Bentonite-based hydrogel materials	91.75	^42^
Activated carbon prepared from *Calotropis gigantea*	92	^43^

#### Effect of pH on adsorption process

**Figure 7:**
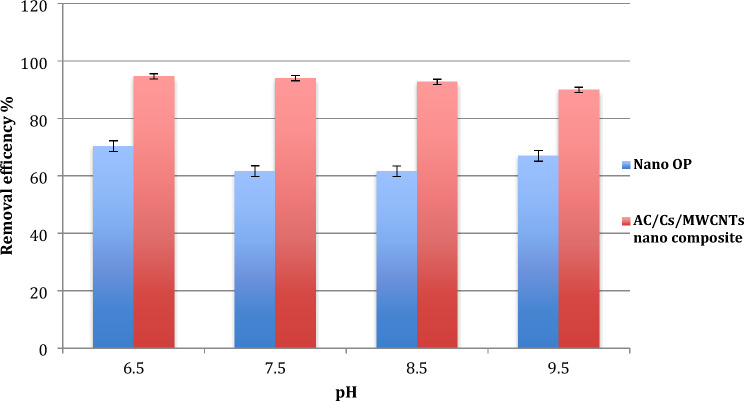
Effect of initial pH of RB dye on adsorption capacity of (**a**) nano OP and (**b**) AC/Cs/MWCNTs nano composite.

pH is considered as one of the most pivotal elements influencing the effectiveness of the adsorption process as well as the solubility of RB dye and the adsorbent total charge being considered. Additionally, the environment acidity is also Considered a powerful influencer for the capability of the competition between hydrogen ions and the dye on disposal centers on the surface of the adsorbent. In this work, pH effect was studied over the range 6.5–9.5 at prior mentioned conditions (Fig. 7). This due to that pH values less than 6.5 causes dye color disappearance. For nano OP and AC/Cs/MWCNTs nano composite, the lower the adsorbent removal efficiency, the higher the pH value up to 9.5. It is noticed clearly that the maximum value of adsorption at the lowest pH 6.5 for the two adsorbents, this is most likely due to protolytic equilibria where a less reactive RB dye species forms. Also, RB dye is water partially soluble at pH lower than 6.0.

#### Effect of temperature on adsorption process

**Figure 8:**
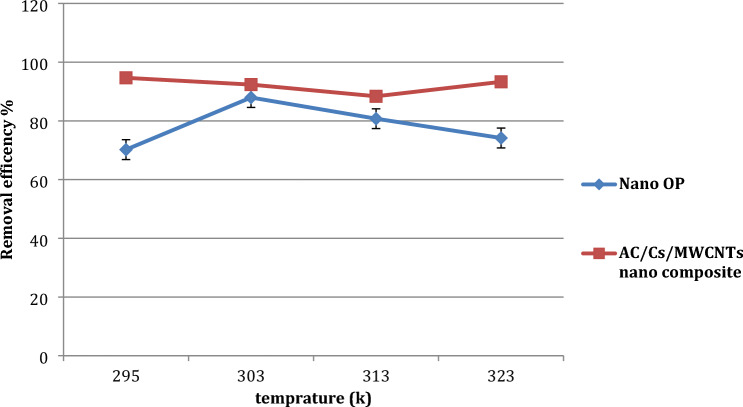
Effect of temperature on adsorption capacity of (**a**) nano OP and (**b**) AC/Cs/MWCNTs nano composite.

Variation in the removal efficiency of RB dye on both nano OP and AC/Cs/MWCNTs nano composite materials with change in temperature was studied by conducting different sets of experiments at different temperatures, that is, 295, 303, 313, and 323 K (Fig. 8). The removal percent of RB dye onto the nano OP was the 70.2, 87.9, 80.7 and 74.16% respectively from 301 to 323 K. and for AC/Cs/MWCNTs nano composite was 94.66, 92.36, 88.38 and 93.3% respectively from 301 to 323 K. The temperature values can be sorted into two domains. The first domain from T = 303 K to T = 313 K about what adsorption efficiency increases in case of nano OP whereas decrease in case of AC/Cs/MWCNTs nano composite. The second domain is from T = 313 K to T = 323 K, adsorption efficiency decreases in case of nano OP whereas increase in case of AC/Cs/MWCNTs nano composite. The active sites accessible for adsorption increases with temperature increasing is the reason behind increasing of adsorption efficiency with temperature increasing. Hence, adsorption of RB by nano OP and AC/Cs/MWCNTs nano composite appears to be an exothermic phenomenon, this also investigated by ΔH values in thermodynamic studies. It is notable that the increasing in temperature makes the pore size greater, as well as increasing the dye diffusion, this is caused by decreasing the viscosity of the solution, which enhances their accessibility to the adsorption on difficult to access sites. Conversely, temperature increasing over 313 K may results in the cell biomass structure becomes weak and the biomolecules exposed to decomposition which is probably due to that the surface active sites become lower and likewise, capacity of the adsorption decreased. The practical results showed that the moderate temperature is the homeopathic value of the adsorption process, these values is 303 K for nano OP and 295 K for AC/Cs/MWCNTs nano composite. This may be interpreted as being more active adsorbent sites at moderate temperature. It is noted that the exothermic nature of RB dye adsorption on nano OP and AC/Cs/MWCNTs nano composite adsorbents will be investigated in a more exhaustive discission through the thermodynamic studies.

### Adsorption isotherm

The two most common adsorption isotherms models used in this regard are the Freundlich and Langmuir. Accordingly, the data of this study have been analyzed in accordance with these both isotherm models. It should be noted that the equilibrium adsorption isotherms of RB dye on nano OP and AC/Cs/MWCNTs nano composite were investigated at temperature of 301 K. The linear form of Freundlich equation was fitting to the excremental data signified by (Eq. 2) and Langmuir represented by Eqs. (3, 4) isotherm models. The constants of both Freundlich and Langmuir equations are shown in Table 4.

**Table 4:** Adsorption isothermal parameters for RB dye on nano OP and AC/Cs/MWCNTs nano composite.

Freundlich parameters			
Adsorbent	n	K_f_ (mg/g)	R_2_
Nano OP	4.2069	4.5087	0.9766
AC/Cs/MWCNTs nano composite	11.3340	4.7691	0.9859

Langmuir parameters			
Adsorbent	Q_max_ (mg/g)	K_L_ (L/mg)	R_L_
Nano OP	3.5038	4.7317	0.0363
AC/Cs/MWCNTs nano composite	4.6707	26.7625	0.00664

Freundlich equation in its Linear form is written as:

2 $$\mathrm{log}{q}_{e}= \frac{1}{n} \mathrm{log}{c}_{e}+ \mathrm{log}{k}_{f}.$$

Langmuir equation in its Linear form is written as:

3 $$\frac{{c}_{e}}{{q}_{e}}= \frac{1}{{k}_{l} {q}_{max}}+ \frac{{c}_{e}}{{q}_{max}}.$$

The separation factor (R_L_) is also calculated for its importance as:

4 $${R}_{L}=\frac{1}{1+{K}_{L}{C}_{0}},$$

where, (q_e_) is the amount of dye which adsorbed at equilibrium (mg/g), (C_e_) is the concentration at equilibrium (mg/L). (K_f_) is the Freundlich constant. This constant is related to the adsorption capacity of the adsorbents (mg^1−(1/n)^ L^1/n^/g). The k_F_ values (mg/g) is determined from the intercept of the Freundlich linear plots. The (1/n) value express the Freundlich-exponent which is related to the intensity of the adsorption process, this value is computed from the slope of the linear Freundlich plots (Eq. 2).

**Figure 9:**
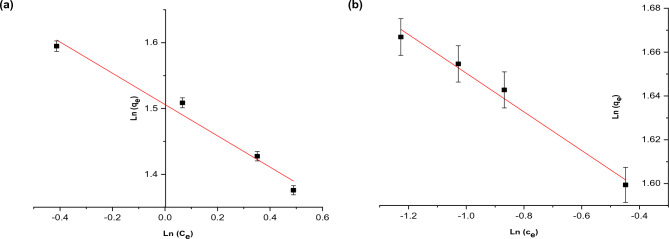
Freundlich isotherm plot of (**a**) nano OP and (**b**) AC/Cs/MWCNTs nano composite.

**Figure 10:**
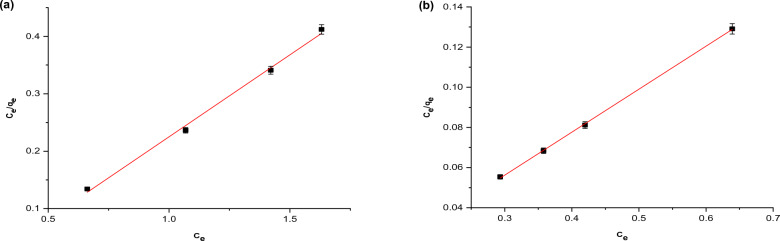
Langmuir isotherm plot of (**a**) nano OP and (**b**) AC/Cs/MWCNTs nano composite.

Where (q_max_) is is the maximum capacity of the adsorption for the adsorbent (mg/g) and (K_L_) is the Langmuir constant related to energy of adsorption (L/mg), and (C_0_) refers to sorbate initial concentration (mg/g). (K_L_) and (q_max_) can be determined from the slope and intercept values of 1/q_e_ versus 1/c_e_ linear plot. The separation factor (R_L_) was also calculated to find out the favorability of the adsorption system. Besides, R_L_ give a description of the type of the Langmuir isotherm. If R_L_ < 1, it implies that adsorption process is favorable adsorption. whilst, R_L_ > 1 it's a sign of unfavourability of the adsorption process. R_L_ can be determined in the temperature ranging (301–323 K) for RB dye, using the relationship mentioned in (Eq. 4). The value of (n) in Freundlich (Eq. 2) is (4.2 and 11.3) for RB dye on both nano OP and AC/Cs/MWCNTs nano composite, respectively. The value of (k_F_) was also (4.5 and 4.7) of both nano OP and AC/Cs/MWCNTs nano composite, respectively, signifies that the experimental conditions are fertile environment for favorable sorption. According to the above discussion it is apparent that the experimental data of RB dye adsorption on nano OP and AC/Cs/MWCNTs nano composite is well fitted by the isotherms as shown in Figs. 9 and 10^6^. Conspicuously, the Langmuir equation is the best fitting considering R^2^ values (0.9962 and 0.99958) for nano OP and AC/Cs/MWCNTs nano composite respectively (Fig. 10) compared with Freundlich equation (0.9766 and 0.9859) for nano OP and AC/Cs/MWCNTs nano composite respectively (Fig. 9). Clearly, the Langmuir equation provided better fitting in terms of R^2^ values. The Langmuir model provides a precise description to the relationship between the amount of adsorbed RB dye and the equilibrium concentration in solution. Is expected that, the adsorption of RB dye occurs on specific homogeneous sites of monolayer adsorption kind with no interaction between the molecules of RB dye and the surface of the solid. The values of q_max_ are (3.5 and 4.6 mg/g) of nano OP and AC/Cs/MWCNTs nano composite, respectively, while the values of (K_L_) (4.7 and 26.7 mol L^−1^) of nano OP and AC/Cs/MWCNTs nano composite, respectively for RB dye are determined through the plot’s intercepts. Separation factor values (R_L_) are (0.036 and 0.0066) of the nano OP and AC/Cs/MWCNTs nano composite, respectively giving obvious evidence to the great favorability of the adsorption operation at solution temperature values under study.

### Kinetic modeling

Table 5 shows the corresponding kinetic parameters obtained by the three models applied in this study. The obtained experimental data of RB adsorption on both nano OP and AC/Cs/MWCNTs nano composite was applied in terms of pseudo first order, pseudo second order, intra particle diffusion and liquid diffusion film models.

**Table 5:** Kinetic parameters for RB dye on nano OP and AC/Cs/MWCNTs nano composite.

Pseudo first-order model						
Adsorbent	Temp. K	K_1_ (min^−1^)	qe, 1, cal (mg/g)	t^0.5^	R^2^	
Nano OP	295	0.03803	1.2504	18.2263	0.8453	
	303	0.06113	2.9496	11.3389	0.9432	
	313	0.02756	0.5609	25.1504	0.8458	
	323	0.01361	0.7373	50.92.92	0.9320	
AC/Cs/MWCNTs nano composite	295	0.0381	1.6928	18.1928	0.9832	
	303	0.0336	1.0994	20.6293	0.9386	
	313	0.0270	1.1426	25.6721	0.7515	
	323	0.0133	0.5402	52.1163	0.8281	

Pseudo second-order model						
Adsorbent	Temp. K	K_2_ (g/mg min)	qe, 2, cal (mg/g)	h (mg/g min)	t^0.5^	R^2^
Nano OP	295	0.1028	3.9945	1.6402	1.7686	0.9982
	303	0.0407	5.1808	1.0937	4.4672	0.9978
	313	0.3287	4.4815	6.6028	0.5531	0.9999
	323	0.2233	3.9556	3.4946	0.8142	0.9995
AC/Cs/MWCNTs nano composite	295	0.0819	5.3304	2.3289	2.22	0.9989
	303	0.1419	5.1557	3.7742	1.2813	0.9996
	313	0.1256	4.8813	2.9935	1.4475	0.9989
	323	0.3744	5.0597	9.5868	0.4856	0.9997

Intraparticle diffusion model						
Adsorbent	Temp. K	K_int_ (mg/g min^−0.5^)	C (mg/g)	R^2^		
Nano OP	295	0.2192	2.1691	0.8215		
	303	0.3978	1.7469	0.8031		
	313	0.0973	3.6957	0.6875		
	323	0.0675	3.3170	0.9341		
AC/Cs/MWCNTs nano composite	295	0.2173	3.4045	0.8850		
	303	0.15622	3.8165	0.8684		
	313	0.1787	3.3621	0.8214		
	323	0.05716	4.5622	0.8107		

Liquid film diffusion model						
	Temp. K	K_fd_	C (mg/g)	R^2^		
Nano OP	295	0.0380	1.1522	0.8453		
	303	0.0611	0.5130	0.9432		
	313	0.0275	2.0867	0.8458		
	323	0.0136	1.7321	0.9320		
AC/Cs/MWCNTs nano composite	295	0.03811	1.1404	0.9832		
	303	0.0336	1.5479	0.9386		
	313	0.0270	1.4660	0.7516		
	323	0.0133	2.2704	0.8282		

**Figure 11:**
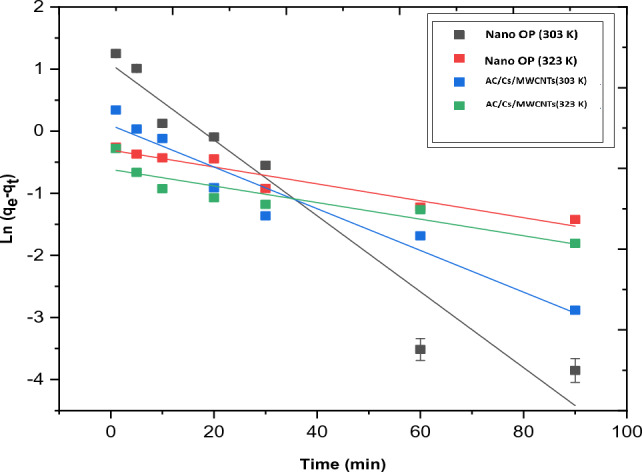
Presentation of the first order plot for RB dye adsorption onto nano OP and AC/Cs/MWCNTs nano composite.

The pseudo first order kinetic equation:

5 $$\mathrm{log}\left({q}_{e}- {q}_{t }\right)= \mathrm{log}{q}_{e,1,cal}- {k}_{1}t,$$

whereas, q_t_ is the amount of solute adsorbed for each unit weight of adsorbent (mg/g) at t (min), q_e_ are the amount of solute adsorbed for each unit weight of adsorbent (mg/g) at equilibrium. k_1_ (min^−1^) is the rate constant of pseudo first order, it can be determined through the plot of log (q_e_ − q_t_) versus t (Fig. 11).

The pseudo second order equation:

6 $$\frac{t}{q}= \frac{1}{{k}_{2}{q}_{e,2}^{2}}+\frac{1}{{q}_{e,2}}t.$$

**Figure 12:**
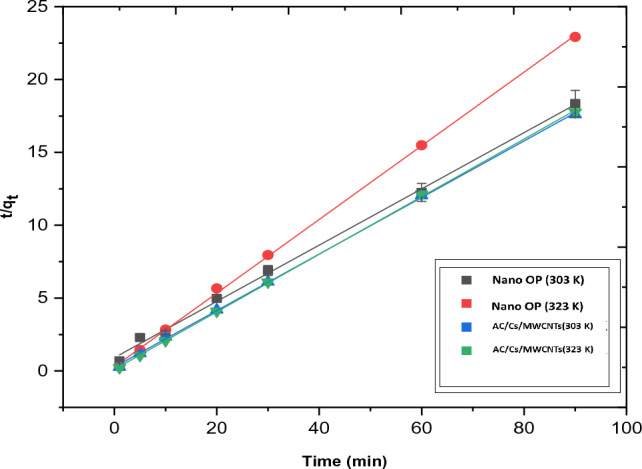
Presentation of the second order plot for RB dye adsorption onto nano OP and AC/Cs/MWCNTs nano composite.

In this equation, K_2_ is the of pseudo second order sorption rate constant (gm mmol^−1^ min^−1^), q_e_ is the amount of soluted sorbate at equilibrium (mmol/g) while, q_t_ is the amount of soluted sorbate on the surface of the adsorbent at time t (mmol/g). k_2_ and q_e_ can be obtained through the intercept and slope of t/qt versus t plot (Fig. 12).

7 $$h= {k}_{2}{q}_{e,2}^{2}$$

where the constant h (mmol g^−1^ min^−1^) provides the value of the initial adsorption, this constant can be experimentally detected through the plot of t/q versus t (Fig. 12).

Intra article diffusion model:

The intraparticle diffusion equation is written as:

8 $${q}_{t}= {k}_{i} {t}^{0.5}+c$$

where, k_i_ is the rate coefficient of the intraparticle diffusion (mg g^−1^ min^−0.5^) and c (mmol g^−1^) provides an idea about the thickness of the boundary layer. The slope and intercept of the linear plot of q_t_ against t^0.5^ give a clear determination for both k_i_ and c.

Liquid diffusion film model:

Liquid diffusion film equation is written as:

9 $$\mathrm{ln }(1 -\mathrm{ F}) = -\mathrm{kfdt }+\mathrm{ C}$$

where F is the fractional attainment of equilibrium (F = q_t_/q_e_), K_fd_ is liquid film diffusion constant. A linear plot of ln (1 − F) versus t with zero intercept would suggest that the kinetics of the sorption process was controlled by diffusion through the liquid surrounding the solid sorbent. q_e_ is the adsorption capacity of the sorbent at equilibrium (mg g^−1^).

In this work, the obtained results of k_1_ values for RB dye at 295 K were 0.03803 and 0.0381 min^−1^ for nano OP and AC/Cs/MWCNTs nano composite respectively, whereas, the R^2^ values were 0.8453 and 0.9832 for nano OP and AC/Cs/MWCNTs nano composite respectively. Also, there is a visible difference in q_e_ values obtained from the plot and those determined experimentally. Based on the above, the pseudo first order model is not considered the appropriate model for perception the RB dye kinetics. The RB dye is grabbed on the active sites present on both nano OP and AC/Cs/MWCNTs nano composite surfaces via chemical bond formation. Under the terms of the Pseudo second order model, the number of the active sites existed on the surface of the adsorbent is the determination factor of the adsorption rate. The values of k_2_ for nano OP and AC/Cs/MWCNTs nano composite adsorption were 0.1028 and 0.0819 g/mg. min at 295 K to RB dye, respectively. The values of regression coefficient for nano OP and AC/Cs/MWCNTs nano composite were 0.9982 and 0.9989 respectively at 295 K. In this case, result obtained through the pseudo second order model indicates that this model give high correlation coefficient (R^2^ > 0.99). Thus, that the pseudo-second-order kinetic model gives the best correlation for the adsorption of RB dye on the nano OP and AC/Cs/MWCNTs nano composite. In addition, in this model there is a great resemblance between the calculated and experimental values of q_e_ for RB dye adsorption by nano OP and AC/Cs/MWCNTs nano composite. subsequently, pseudo second order model is the most ideal model to describe the kinetic attitude of RB dye adsorption. The great portability of the pseudo second order model for this kinetic data at all temperatures is agree with other studies results^44^.

#### Adsorption mechanism

**Figure 13:**
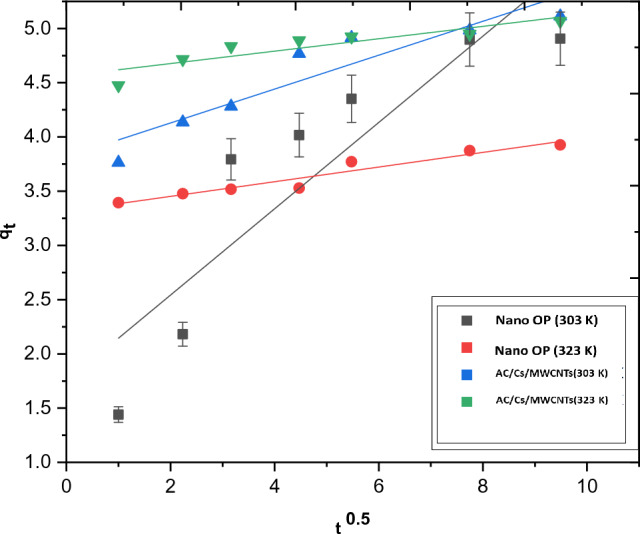
Presentation of intraparticle diffusion model for RB dye adsorption onto nano OP and AC/Cs/MWCNTs nano composite.

Generally, the adsorption mechanism of a given adsorbed solute onto an adsorption surface and deep penetration can be thought to proceed through the diffusion of the solute particles into the adsorbent pores, followed by a linking between the adsorbent active sites and the solute entity. The rate determining step in such adsorption process can be located using the intraparticle diffusion model, given by Weber and Morris (Eq. 8)^45^ where, k_ipd_ can be extracted from plot slope q_t_ versus t^0.5^. To obtain the rate determining step of an adsorbing system, the drawing straight line has to cross the origin and the value of intercept. For an adsorbing system the straight line should pass through the origin and the intercept value affords a clue about the deviancy from model of intraparticle diffusion or involvement of the film diffusion mechanism^46^. In Fig. 13, the value of k_ipd_ for nano OP and AC/Cs/MWCNTs adsorption were observed to be 0.3978 and 0.15622 mg/g min^0.5^ at 303 K. Thus, straight line was obtained without origin crossing, it showed clear intercepts of 1.7469 and 3.8165 at 303 K. This may suggest that, the intraparticle diffusion is not the main supporting mechanism for RB dye adsorption.

**Figure 14:**
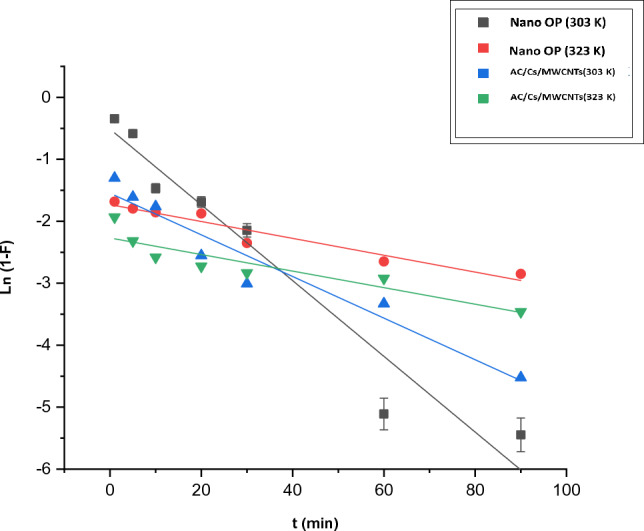
Presentation of liquid film diffusion model for RB dye adsorption onto nano OP and AC/Cs/MWCNTs nano composite.

In the adsorption process where the rate controlling step is the diffusion through a liquid film, the mechanism of transporting the adsorbate to the adsorbent proceeds through the solid–liquid interface that can be addressed by the liquid film diffusion model. In such case, the thickness of the liquid film around the particle of adsorbent will control the rate of diffusion, (Eq. 9)^47^. In the current work, k_fd_ for nano OP was 0.0611 g/g min and have an intercept at 0.5130, where that for AC/Cs/MWCNTS gave 0.0336 g/g min with intercept of 1.5479 (Table 5). In Fig. 14, the absence values of non-zero intercept, designated the mechanism of dye adsorption to be of film diffusion one. The encountered small deviation could be accounted to the shear originated via agitation, resulted in considerable aqueous layer-thickness reduction on particles. Therefore, the limiting step in the adsorption of RB dye might be assigned to the resistance of the boundary-layer or the film diffusion.

### Parameters of thermodynamics

Parameters such as enthalpy ΔH (kJ mol^−1^), entropy ΔS (J mol^−1^ K^−1^) and standard free energy of activation ΔG (kJ mol^−1^) were thermodynamically investigated (Table 6) to estimate the thermodynamic viability and spontaneous behavior of the adsorption process. These all obtained by applying the following equations:

10 $$\Delta \mathrm{G}= \Delta \mathrm{H}- T\Delta S$$

11 $$\Delta \mathrm{G}= -RT ln{k}_{d}\mathrm{ where }{k}_{d}= \frac{{q}_{e}}{{c}_{e}}$$

12 $$\mathrm{log}{k}_{d}= \frac{\Delta S}{2.301 R}- \frac{\Delta H}{2.301 RT}$$

where, T is the temperature in Kelvin, R refers to the gas constant. K_d_ is the standard thermodynamic equilibrium constant, q_e_ is the amount RB dye that adsorbed per unit mass of nano OP and AC/Cs/MWCNTs nano composite at equilibrium (mg g^−1^) and C_e_ is the equilibrium aqueous concentration of RB dye. ΔH and ΔS are detected from the slopes and intercepts of the linear plot of log K_d_ against 1/T (K^−1^). And for more further supporting the confirmation that the adsorption is the prevalent mechanism, of the activation energy (E_a_) and sticking probability (S^*^) values were also determined through the experimental data.

**Table 6:** Thermodynamic parameters for RB adsorption on nano OP and AC/Cs/MWCNTs nano composite.

Adsorbent	Temp. (K)	∆G (kJ/mol)	∆S (J/mol k)	∆H (KJ/mol)	S*	Ea (KJ/mol)
Nano OP	295	− 2.4269	8.2237	− 0.9325	0.3404	1.3320
	303	− 2.4927	–	–	–	–
	313	− 2.5749	–	–	–	–
	323	− 2.6571	–	–	–	–
AC/Cs/MWCNTs nano composite	295	− 2.4645	8.3236	− 9.0552	1.9378	8.3899
	303	− 2.5311	–	–	–	–
	313	− 2.6143	–	–	–	–
	323	− 2.6975	–	–	–	–

13 $${S}^{*}=\left(1-\theta \right)\mathrm{exp} (\frac{{E}_{a}}{RT})$$

All of the ΔH (− 0.9325 for nano OP and − 9.0552 for AC/Cs/MWCNTs nano composite) values were negative, indicating that the process is an exothermic. The negative ΔG at all studied temperatures combined with the positive ΔS give an acceptable suggestion that the sorption reactions are spontaneous with a great affinity and also the presence of low energy barrier of RB dye adsorption processes using both nano OP and AC/Cs/MWCNTs nano composite. The negative enthalpy change (ΔH) values for the RB dye adsorption reaction indicate the exothermic nature of the present reaction. ΔH values obtained from adsorption study of RB dye onto and AC/Cs/MWCNTs nano composite are lower than those onto nano OP. This result for RB dye telling evidence that the interactions between RB dye and the surface groups of the AC/Cs/MWCNTs nano composite may be weaker than those of the surface groups of the nano OP. However, the positive values of E_a_ reveal the presence of an energy barrier in the adsorption process. These results indicated that the adsorption has a slightly higher potential barrier for both nano OP and AC/Cs/MWCNTs nano composite systems and prove that the sorption process is chemisorption nature. The value of S^*^ ≤ 1 (0.3404) for the nano OP and slightly larger than 1 (1.9378) for AC/Cs/MWCNTs nano composite hence the sticking probability of the RB dye onto the two the adsorbent systems are fairly well.

## Conclusion

In this work, adsorption of RB dye was investigated using nano OP and the new prepared composite (AC/Cs/MWCNTs nano composite) as an effective adsorbent. The FT-IR tests showed that the AC/Cs/MWCNTs nano composite is containing many active functional groups compared with nano OP, nano AC and nano AC/Cs. The adsorption experiments were applied to study the effects of different variables including pH, initial concentration of RB, and the temperature. Pseudo-first order, pseudo-second order, intraparticle diffusion and liquid film diffusion kinetic models were applied to study the adsorption process kinetic. The results showed that pseudo-second order and liquid film diffusion kinetic model can control the adsorption process mechanism. Furthermore, Langmuir and Freundlich models were used to consider the equilibrium adsorption. The Langmuir isotherm model was the preferred fitting model for both nano OP and AC/Cs/MWCNTs nano composite. According to the calculated absorption mean free energy (E = 1.3320 and 8.3899) for nano OP and AC/Cs/MWCNTs respectively, it was that reveal the presence of an energy barrier in the adsorption process. In general, AC/Cs/MWCNTs nano composite revealed a great adsorption efficiency for RB dye compared with nano OP.

## Data Availability

The datasets used and/or analyzed during the current study are available from the corresponding author upon reasonable request.
